# Developing implementation strategies for promoting integrative oncology outpatient service delivery and utilisation: a qualitative study in Hong Kong

**DOI:** 10.3389/fpubh.2024.1414297

**Published:** 2024-08-30

**Authors:** Leonard Ho, Ming Hong Kwong, Angus S. C. Li, Per Nilsen, Fai Fai Ho, Claire C. W. Zhong, Charlene H. L. Wong, Wai Ling Lin, Vincent C. H. Chung

**Affiliations:** ^1^Jockey Club School of Public Health and Primary Care, Faculty of Medicine, Chinese University of Hong Kong, Shatin, Hong Kong SAR, China; ^2^Department of Medicine, Health and Caring Sciences, Linköping University, Linköping, Sweden; ^3^School of Chinese Medicine, Faculty of Medicine, Chinese University of Hong Kong, Shatin, Hong Kong SAR, China

**Keywords:** cancer, integrative medicine, implementation science, delivery of health care, qualitative research

## Abstract

**Purpose:**

Current evidence supports the use of integrative oncology (IO) interventions in cancer supportive care. The demand for outpatient IO services in Hong Kong is expected to soar following the surge in cancer incidence due to population ageing. This study identified the factors influencing the delivery and utilisation of outpatient IO from local stakeholders’ perspectives and developed corresponding implementation strategies.

**Methods:**

This study involved two sequential stages. First, with individual semi-structured interviews guided by the Theoretical Domains Framework (TDF), we explored stakeholders’ views on the barriers to and facilitators for implementing IO. Second, guided by a TDF-based qualitative data analysis of interview transcripts, we performed intervention mapping to develop Behaviour Change Wheel-based implementation strategies that may overcome the barriers and strengthen the facilitators.

**Results:**

We interviewed 31 stakeholders, including traditional Chinese medicine (TCM) practitioners (*n* = 8), biomedically-trained doctors (*n* = 7), nurses (*n* = 6), administrators (*n* = 4), caregivers (*n* = 4), and pharmacists (*n* = 2). The key local factors influencing outpatient IO are (1) lacking nursing and administrative workforce supporting IO service delivery, (2) lacking awareness of IO services among healthcare professionals, administrators, patients, and caregivers, and (3) lacking knowledge among healthcare professionals of herb–drug interaction and herbal toxicities.

**Conclusion:**

We recommended a multi-faceted implementation strategies package that included arranging funding to train, recruit, and retain nursing and administrative staff, devolving resources into promoting interprofessional collaborations and evidence on IO effectiveness and safety, integrating evidence on herb–drug interactions and herbal toxicities into automated electronic health record systems monitored by pharmacists with dual qualifications in TCM and conventional pharmacy.

## 1 Introduction

Many healthcare organisations worldwide have started providing integrative oncology (IO) services as part of their routine cancer care (1). As a sub-field in integrative medicine, IO is a patient-centred, evidence-informed approach that utilises traditional and complementary medicine (T&CM) alongside conventional cancer treatments (2), aiming to optimise the health, quality of life, and clinical outcomes of cancer patients, in addition to empowering them to become active participants in their care before, during, and beyond cancer treatment (2). Traditional Chinese medicine (TCM) is one of the major modalities of T&CM popular amongst Chinese populations worldwide. It is a comprehensive system that originated in ancient China that involves the study of life, health and diseases and is applied to health preservation and disease prevention, diagnosis, and treatment, as well as rehabilitation (3). Chinese herbal medicine and acupuncture are the most common TCM interventions. The former refers to the usage of natural medicinal ingredients (i.e., plants, animals, and minerals) and their processed products for therapeutic purposes (4), and the latter refers to using stainless-steel filiform needles to puncture specific “acupoints” on the body to trigger specific therapeutic effects (5). Evidence supporting the effectiveness of TCM in IO has been systematically synthesised. For instance, overviews of reviews found that Chinese herbal medicine can improve the quality of life among patients receiving cancer palliative care (6–8). Acupuncture may also alleviate cancer-related fatigue, chemotherapy-induced nausea and vomiting, and leukopenia (9, 10). The co-administration of herbal medicine (including Chinese herbal medicine) and chemotherapeutic agents is often discouraged by clinical oncologists due to the potential of undesirable herb–drug interactions and herbal toxicities. However, evidence on herb–drug interactions is largely extrapolated from mechanistic research and may not necessarily be translatable to clinical practice (11), while causality between herbal medicine and herbal toxicities was supported by patient recall, clinical suspicion, exclusion, and algorithms (12). Heavy metal contamination might further confound the findings regarding herbal toxicities (13).

TCM has been playing a pivotal role in filling the effectiveness gaps of conventional medicine (14), providing a substantial portion of chronic disease management services in Hong Kong outpatient settings (15). The private sector is the main provider of TCM services. In the public sector, the government established 18 Chinese Medicine Centres for Training and Research (CMCTRs) to provide a relatively small volume of service. Aiming to facilitate training placements for local TCM graduates, each of these CMCTRs is operated through a tripartite collaboration between the Hospital Authority (the statutory body providing most tax-funded healthcare services in Hong Kong), a non-governmental organisation, and a local university (16). Some non-governmental organisations and registered charities are also providers of TCM outpatient services.

In Hong Kong, TCM was not introduced into inpatient settings until 2014 when the Hospital Authority initiated the “Integrated Chinese–Western Medicine” (ICWM) pilot programme, providing stroke rehabilitation, musculoskeletal pain management, and cancer palliative care services in public hospitals (17). The programme aims to offer affordable IO services to patients in inpatient settings and encourage collaborations between TCM practitioners (TCMPs) and biomedically-trained doctors (BMDs) through co-formulating and executing evidence-based patient management plans (18). However, ICWM services are mainly confined to inpatient care, and their collaboration experience may not be directly transferable outside the wards. The demand for outpatient IO services in Hong Kong is expected to soar following the projected increase in annual cancer incidence to more than 42,000 in 2030 (19). In response to patients’ demand for IO (20) and an international trend of providing cancer supportive care in outpatient settings (21), outpatient IO services emerged in the private sector. In Hong Kong, interprofessional collaborations must rely on the personal networks between private TCMPs and BMDs as there is no established interprofessional collaboration platform (22). Also, without formal implementation strategies to support shared care, collaboration between TCMPs and BMDs remains challenging, despite the availability of evidence supporting the benefits of IO (23).

Empirical models have been proposed to streamline outpatient IO service delivery with central coordinators (24), but how such models work for bringing together TCM and conventional medicine service is unclear. We conducted a mixed-methods systematic review to synthesise 28 empirical studies on the factors influencing IO service delivery in conventional cancer care settings (including inpatient and outpatient) around the world (25). Supported by evidence from Europe, North America, and Oceania, this study recognised three major implementation barriers, including the lack of IO knowledge, the limitation on funding, and the low level of IO receptiveness among healthcare professionals, and three major implementation facilitators, including the dissemination of clinical evidence on IO, the upskilling of professionals on IO service delivery, and the availability of supportive organisational climate. Despite the international evidence, it is uncertain how these lessons learnt overseas should/could be translated into implementation strategies relevant to the Hong Kong healthcare system context. Despite the international evidence on service delivery models and implementation, it is uncertain how these lessons learnt overseas should/could be translated into implementation strategies relevant to the Hong Kong healthcare system context.

To be comprehensive, the factors influencing outpatient IO service delivery and utilisation among potential stakeholders must also be investigated based on established implementation science theories, models, or frameworks. Such studies would allow theory-guided assessment of those factors across service providers (e.g., BMDs, TCMPs, nurses, administrators, and pharmacists) and caregivers to inform the formulation of theory-informed implementation strategies to support implementation. These strategies would then assist policy-makers and authorities in promoting outpatient IO service delivery and utilisation in the future. Implementation strategies constitute the “how to” of integrating evidence-based interventions, programmes, and other practices into routine use in healthcare and other settings (26). An implementation strategy has been defined as “a systematic intervention process to adopt and integrate” evidence-based practices into usual care (27).

Conducted within the Hong Kong healthcare system context, this study aims to (1) identify the factors influencing the delivery and utilisation of outpatient IO services from the perspectives of service providers and caregivers; and (2) develop theory-informed implementation strategies for promoting the delivery and utilisation of outpatient IO services, addressing identified factors at behavioural, organisation, and policy levels.

## 2 Methods

**Figure 1:**
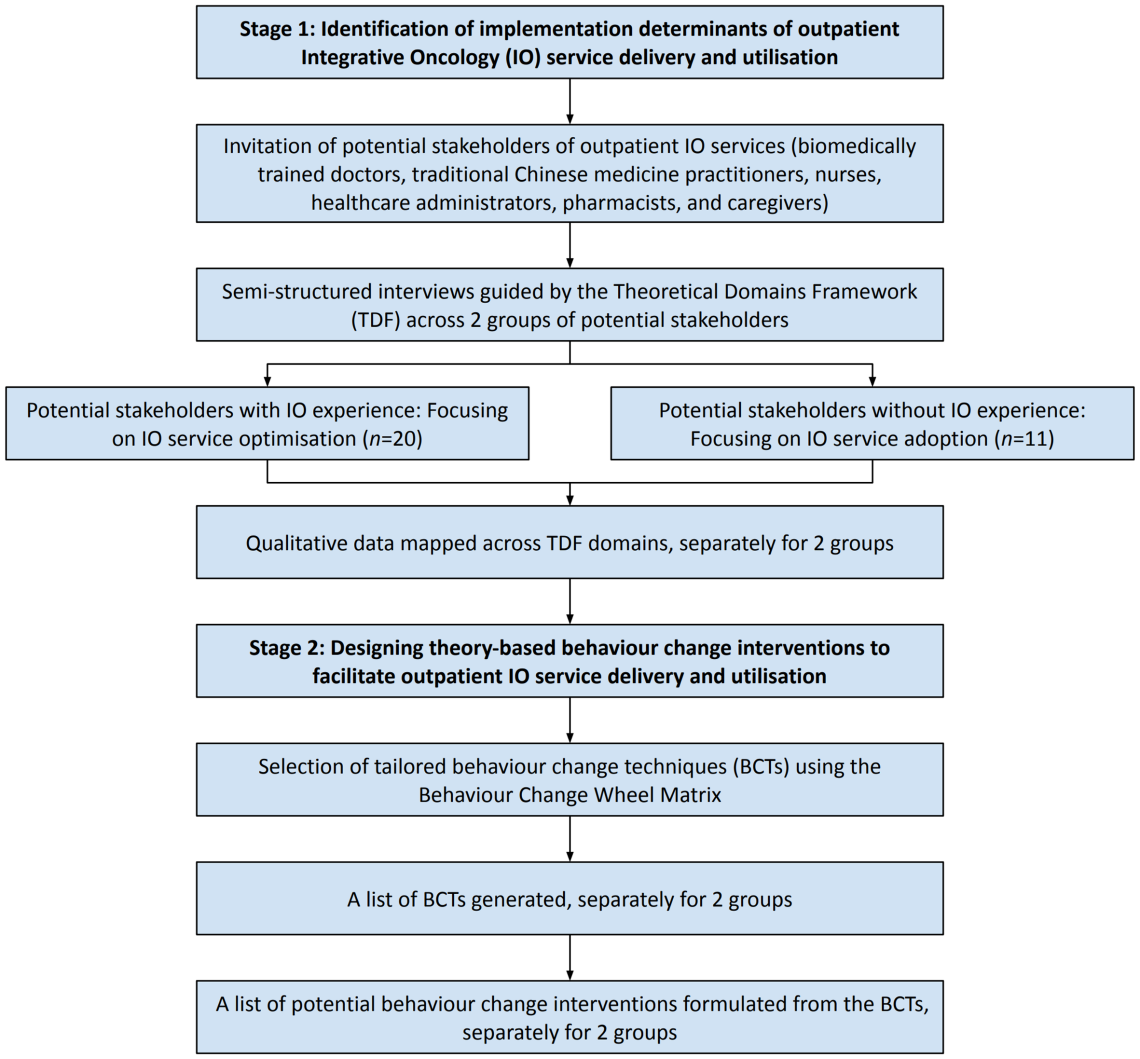
Overview of the two-stage study for formulating implementation interventions.

The study consists of two sequential stages. In stage 1, we explored the views of different stakeholders on the potential factors influencing the delivery and utilisation of outpatient IO services. Guided by the Theoretical Domains Framework (TDF) (28) and the Behaviour Change Wheel (BCW) (29), qualitative data collected from the semi-structured virtual interviews provided insights into stakeholders’ perspectives on capacities, opportunities, and motivations for delivering and utilising outpatient IO services. In stage 2, we performed intervention-mapping to establish theory-informed implementation strategies that address the factors we identified in stage 1. Figure 1 provides an overview of this two-stage study. We obtained ethical approval from the Survey and Behavioural Research Ethics Committee of the Chinese University of Hong Kong before the commencement of interviews (Reference number: SBRE-21-0164).

### 2.1 Stage 1: identification of factors influencing outpatient IO service delivery and utilisation

#### 2.1.1 Participant sampling

The semi-structured individual virtual interviews involved two groups of potential stakeholders of outpatient IO services. We planned to include at least 15 healthcare professionals (e.g., BMDs, TCMPs, nurses, and healthcare administrators) and caregivers in the first group. Those healthcare professionals must have at least 5 years of experience delivering IO services (e.g., via the ICWM programme) in Hong Kong, while the caregivers must have experience caring for cancer patients who utilised local IO or ICWM services. We planned to involve in the second group with at least 10 healthcare professionals without previous experience delivering IO services. The purposive sampling of participants with or without IO experience allowed us to investigate implementation challenges during the scaling up or maintenance of existing services, or during the establishment of new services, respectively. We sampled more interviewees in the experienced group as they are supposed to be more informed regarding IO implementation. The target sample size of 25 (at least 15 with IO experience plus at least 10 without IO experience) fulfilled the number of participants expected to achieve saturation in implementation research (*n* = 16–24) (30, 31).

#### 2.1.2 Data collection

For the two groups of interviewees, we prepared separate interview guides based on the TDF (Supplementary Boxes S1–S4) (28). This framework contains 14 theoretical domains generated from 128 constructs based on 33 health and social psychology theories on behaviour change (28). These domains provided comprehensive coverage of the complex factors of behaviours that influence the implementation of outpatient IO services at the individual, organisational, and societal levels.

We first asked the healthcare professionals to describe: (1) the activities to promote IO service delivery that had been useful (for those with experience) or were expected to be useful (for those without experience) and (2) the specific incidences when they delivered IO services but found that the experience was not satisfactory (for those with experience) or where the experience was expected to be unsatisfactory (for those without experience). These two questions were followed by specific prompts relating to the possible influences of implementing outpatient IO services, covering all TDF domains. For caregivers, we inquired about their expectations or experience of assisting patients in accessing outpatient IO services.

The interviews were hosted by authors with expertise in qualitative interview methods (MHK and ASCL) and IO in Hong Kong (VCHC and LH). Each interview took approximately 1 h and was conducted on virtual meeting platforms between February 2022 and July 2022. Each interview was audio-recorded after receiving written informed consent from the participant.

#### 2.1.3 Data analysis

For stage 1, we performed interviews, transcription, and data analysis concurrently to track emerging themes, which enabled timely follow-up of questions for the next interview or clarifications with the interviewees. We transcribed the audio recordings verbatim and imported the transcripts into NVivo for data analysis in three phases as follows.

##### 2.1.3.1 Phase 1: coding of transcripts

The responses from the first 12 participants were coded independently by two authors (ASCL and MHK). Specifically, the coding involved (1) reading the participants’ responses in the transcripts, (2) considering their relevance to the definitions of the TDF domains or constructs, and (3) assigning these responses to one or more domains via directed content analysis (32). The coders then compared their coding results and developed a consensus-based coding scheme. This approach ensured coding consistency as this consensus-based coding scheme guided subsequent coding.

The authors coded the remaining interviews and met frequently (after completing the coding of every five interviews) to assess consistency and seek consensus. When the authors could not reach a consensus on coding, they would discuss with a senior author (VCHC or PN) on text interpretation. If the authors could not reach an agreement on assigning a code to a single domain, they would assign the code to all the domains they identified.

##### 2.1.3.2 Phase 2: generating themes on the factors influencing outpatient integrative oncology service delivery and utilisation

Following current guidelines (33), after coding the data into theoretical domains, one author was responsible for generating themes attributable to outpatient IO service delivery and utilisation inductively from the codes via thematic analysis. Another author cross-checked and confirmed the themes and the codes. When they could not reach a consensus, the discrepancy was resolved via discussion among the authors or by arbitration of senior authors (VCHC or LH).

The themes were then subjected to a rating process by all the authors involved in the coding process. The team used a deliberated consensus process to assign a rating to each theme within each domain, across the two groups of participants, based on the criteria reflecting its strength (degree of emphasis by the participants in that group) and valence (positive or negative influence) (34). The ratings ranged from −2 to +2, where −2 refers to a strong negative influence on implementation and +2 a strong positive influence. Next, we selected for further discussion the themes that had a strong positive or negative influence on outpatient IO implementation and were identified as key themes in the mentioned mixed-methods systematic review of international experiences on IO implementation in cancer care settings (denoted as “key factors identified in Hong Kong and overseas”) (25). Meanwhile, the themes that had strong a positive or negative influence on outpatient IO implementation but were not mentioned in the systematic review were also chosen for further discussion (denoted as “key factors identified only in Hong Kong”).

### 2.2 Stage 2: identifying behaviour change techniques and designing theory-informed implementation strategies to facilitate outpatient integrative oncology service delivery and utilisation

The research team for this stage consisted of authors with expertise in qualitative research methods (MHK), integrative medicine in Hong Kong (VCHC and LH), and implementation research (PN and CCWZ). We applied our stage 1 TDF-based findings to the three core components of the BCW necessary to induce a target behaviour: (1) capability, (2) opportunity, and (3) motivation (COM–B). Using the COM–B model and the TDF, we performed a behaviour diagnosis to determine what needs to be changed to enable outpatient IO service delivery or utilisation.

Having identified the relevant COM-B components, we had thorough discussions on how to address each factor through particular intervention functions based on the Michie et al. matrix of links between COM-B and intervention functions and related comments from the interviewees (29). After choosing the intervention functions deemed suitable by the team, we considered the policy categories most applicable to supporting the identified intervention functions based on the Michie et al. matrix of links between intervention functions and policy categories (29).

Subsequent to the identification of intervention functions and policy categories, we proceeded to identify behaviour change techniques (BCTs) using the BCT Taxonomy v1 (BCTTv1) (35). Mapped across the TDF domains, BCTs are the active components to underpin the delivery of the intervention functions under the relevant policy categories, aiming to promote behaviours that enable intervention implementation (29). In this study, they supported the generation of implementation strategies expected to overcome barriers to and strengthen facilitators for the delivery or utilisation of IO services at individual, organisational, and policy levels. After the completion of data analysis for every two to three interviews, we would discuss the BCTs relevant to the identified intervention functions, policy categories, and most importantly, the Hong Kong healthcare system context, and design and/or amend implementation strategies to operationalise the BCTs based on the findings at that time point and adjust the content iteratively until the completion of all interview data analysis.

The above approach ensured the logic of the strategy choice by explicitly linking a given BCT with factors stated by the participants, with concomitant consideration of organisational and policy contexts.

## 3 Results

### 3.1 Participants

We were able to interview a total of 31 participants in this qualitative study, exceeding the number of participants needed to achieve saturation. The first group (with IO experience) consisted of 16 healthcare professionals [i.e., BMDs (*n* = 4), TCMPs (*n* = 4), nurses (*n* = 4), and healthcare administrators (*n* = 4)] and four caregivers. The second group (without IO experience) comprised another 11 healthcare professionals [i.e., BMDs (*n* = 3), TCMPs (*n* = 4), nurses (*n* = 2), and pharmacists (*n* = 2, of which one is dual qualified in Chinese herbal and conventional pharmacy)]. We invited the two pharmacists after completing the first few interviews, where participants expressed that it was essential to consider the views of local pharmacists as they are likely to play a role in IO service delivery. Table 1 presents participants’ professional and demographic characteristics.

**Table 1:** Basic professional and demographic characteristics of participants.

Participant characteristics and demographics	Frequency (%)
Female	15 (48.4)
Age group (years)	
<30	7 (22.6)
31–45	18 (58.1)
46–60	4 (12.9)
>61	2 (6.5)
Role	
Traditional Chinese medicine practitioner	8 (25.8)
Biomedically-trained doctor	7 (22.6)
Nurse	6 (19.4)
Healthcare administrator	4 (12.9)
Caregiver	4 (12.9)
Pharmacist	2 (6.5)
Years of practice or caregiving experience	
<5	10 (32.3)
6–10	6 (19.4)
11–15	9 (29.0)
16–20	4 (12.9)
>21	2 (6.5)
Years of integrative oncology experience as service provider or caregiver (*n* = 20)	
≥5–10	18 (90.0)
11–15	1 (5.0)
16–20	0 (0.0)
>21	1 (5.0)

### 3.2 Key factors influencing outpatient integrative oncology service delivery and utilisation

Qualitative data analysis generated 19 themes of factors, and we mapped all identified themes to at least one of the three components of the COM-B system, as well as 10 of the 14 TDF domains. The key factors identified in Hong Kong and overseas from the 31 interviews were:

- lack of recognition among healthcare professionals, patients, and caregivers on the evidence supporting the safety and effectiveness of IO interventions;
- lack of knowledge among healthcare professionals and healthcare administrators regarding IO; and
- inefficient interprofessional communication and collaboration system.

The key factors identified only in Hong Kong were:

- lack of nursing and administrative workforce supporting IO service delivery;
- lack of awareness of IO services among healthcare professionals, healthcare administrators, patients, and caregivers; and
- lack of knowledge among healthcare professionals regarding herb–drug interaction and herbal toxicities.

Table 2 illustrates all themes summarised from the interviews. Below will describe the six key factors in detail with relevant quotes extracted from the transcripts. The behaviour change techniques used to resolve those key factors will also be discussed.

**Table 2:** Factors influencing the implementation of outpatient integrative oncology services in Hong Kong classified by the Theoretical Domains Framework.

COM–B system	TDF domains	Factors identified from interviews (Themes)	Strength and valence ratings	
			Participants with IO experience	Participants without IO experience
Psychological capability	Knowledge	Lack of awareness of IO services among healthcare professionals, healthcare administrators, patients, and caregivers^#^	−2	−2
		Lack of knowledge among healthcare professionals regarding herb–drug interactions and herbal toxicities^#^	–2	−1
		Lack of knowledge among healthcare professionals, healthcare administrators, and the public regarding IO services^*^	−2	−1
		Lack of content regarding interprofessional collaboration between TCM, conventional medicine, and nursing in undergraduate education	−1	−1
	Memory, attention, and decision processes	Difficulties in decision-making among patients and caregivers on IO service utilisation	0	−1
Physical capability	Skills	Lack of skills among healthcare professionals in delivering IO services	−1	−1
Reflective motivation	Beliefs about consequences	Lack of recognition among healthcare professionals, patients, and caregivers of clinical evidence supporting safety and effectiveness of IO interventions^*^	−2	−2
		Lack of knowledge among healthcare professionals about the potential ethical or legal consequences of collaborative IO service delivery	−1	−1
	Social/professional role and identity	Failure among BMDs and nurses to recognise the compatibility of IO service delivery with their duties and responsibilities	−1	−1
	Intention	Lack of career prospects for TCMPs and nurses involved in IO service delivery	0	−1
Automatic motivation	Reinforcement	Lack of motivation among healthcare professionals in delivering IO service	−1	−1
	Emotion	Negative feelings among BMDs and nurses towards TCM and TCMPs	−1	0
		Feelings of being excluded by the conventional medicine-dominated healthcare system among TCMPs	−1	0
		Lack of emotional understanding among BMDs and nurses towards patients’ requests for IO referral	0	0
Physical opportunity	Environment and resources	Lack of nursing and administrative workforce supporting IO service delivery^#^	−2	−1
		Lack of structured referral mechanisms and clear operation procedures for IO service delivery	−2	0
		Cost of IO services	0	0
Social opportunity	Social influences	Inefficient interprofessional communication and collaboration system^*^	0	0

BMD, Biomedically-trained doctor; COM–B, Capability, Opportunity, Motivation, and Behaviour; IO, Integrative oncology; TCM, Traditional Chinese medicine; TCMP, Traditional Chinese medicine practitioner; TDF, Theoretical Domains Framework.

* Themes that have a strong positive/negative influence on outpatient IO implementation and were identified in a mixed-methods systematic review (22) of international experiences on IO service implementation factors in cancer care settings (i.e., “key factors identified in Hong Kong and overseas”).

# Themes that have a strong positive/negative influence on outpatient IO implementation but were not identified in the mentioned mixed-methods systematic review (i.e., “key factors identified only in Hong Kong”).

#### 3.2.1 Capability (COM-B component)

Overall, six themes were relevant to the capability component of the COM-B system, covering three TDF domains (knowledge; memory, attention, and decision process; and skills) (Table 3). This component included one key factor identified in Hong Kong and overseas and two key factors identified only in Hong Kong.

**Table 3:** Recommended implementation strategies for addressing implementation factors related to capability.

Implementation factors identified from interviews (Themes)	COM–B (TDF domain)	Intervention functions	Policy categories	Behaviour change techniques	Implementation strategies
Lack of awareness of IO services among healthcare professionals, healthcare administrators, patients, and caregivers^#^	Psychological capability (Knowledge)	Education	Communication/marketing	Information about health consequences	Promoting IO services (including information on safety and effectiveness) to the public and elucidating progress of TCM modern development Explaining the regulatory and complaint mechanisms on TCM practice to the public Explaining the fees of IO services, as well as relevant subsidy schemes to patients and caregivers
Lack of knowledge among healthcare professionals regarding herb–drug interactions and herbal toxicities^#^		Enablement	Environmental/social planning	Restructuring the physical environment	Developing an online information platform for herb–drug interactions and Chinese herbal medicine safety for all healthcare professionals Integrate safety information into existing electronic health record systems which will enable automated alert when contra-indication appears
		Enablement	Fiscal measures	Problem solving	Allocating additional resources to improve the research capacity on TCM and IO safety and effectiveness
		Enablement	Service provision	Social support (practical)	Recruiting pharmacists with dual qualifications in TCM and Conventional pharmacy to update the online information platforms for herb–drug interactions and Chinese herbal medicine safety Recruiting pharmacists with dual qualifications in TCM and Conventional pharmacy to provide relevant consultation services on the co-administration of TCM herbs and Conventional drugs
Lack of knowledge among healthcare professionals and healthcare administrators regarding IO^*^		Education	Communication/ marketing	Information about health consequences	Organising continuing professional development programmes on IO services and encouraging all healthcare professionals to participate
		Enablement	Fiscal measures	Problem solving	Allocating additional resources to improve the research capacity on TCM and IO safety and effectiveness
Lack of content regarding interprofessional collaboration between TCM, conventional medicine, and nursing in undergraduate education		Education	Guidelines	Feedback on behaviour	Incorporating BMD–TCMP–nurse interprofessional communication skills training into undergraduate education
Difficulties in decision-making among patients and caregivers on IO utilisation	Psychological capability (Memory, attention, and decision processes)	Education	Communication/marketing	Information about health consequences	Promoting details of IO services (including information on safety and effectiveness) to the public
		Enablement	Regulation	Social support (practical)	Drafting accreditation criteria for the providers (TCMPs and BMDs) of IO services Developing the list of accredited providers (TCMPs and BMDs) of IO services
Lack of skills among healthcare professionals in delivering IO services	Physical capability (Skills)	Training	Guidelines	Instruction on how to perform a behaviour	Planning and delivering sustainable clinical training programmes for IO services with a medium term aim of improving local training capacity and capability
		Training	Service provision	Demonstration of the behaviour	Inviting Chinese Mainland TCM experts to participate in the development of outpatient IO service. Their expertise is expected to enhance practice skills of IO delivery for all healthcare professionals

BMD, Biomedically-trained doctor; COM–B, Capability, Opportunity, Motivation, and Behaviour; IO, Integrative oncology; TCM, Traditional Chinese Medicine; TCMP, Traditional Chinese Medicine practitioner; TDF, Theoretical Domains Framework.

* Themes that have a strong positive/negative influence on outpatient IO implementation and were identified in a mixed-methods systematic review (22) of international experiences on IO service implementation factors in cancer care settings (i.e., “key factors identified in Hong Kong and overseas”).

# Themes that have a strong positive/negative influence on outpatient IO implementation but were not identified in the mentioned mixed-methods systematic review (i.e., “key factors identified only in Hong Kong”).

##### 3.2.1.1 Knowledge

*Key factor identified only in Hong Kong: lack of awareness of IO services among healthcare professionals, healthcare administrators, patients, and caregivers.* In Hong Kong, lacking awareness of IO services was highlighted as one of the key reasons explaining why healthcare professionals and administrators do not deliver or adopt IO services in their daily practice. It might also explain why patients and caregivers do not use or recommend IO services. The common cultural practice of consuming Chinese herbal soups and consulting TCMPs for health maintenance does not necessarily contribute to the establishment or usage of IO services.

*“When we consulted BMDs for the first time, there was no information about IO posted in the oncology clinics, nor there was information provided by the nurses. It is always better to have advertisements or promotion leaflets available for patients because they might think: “Oh, TCM and TCMPs may help!” Perhaps many patients and caregivers are frustrated about not having authoritative information about IO services.”* – a caregiver with IO experience.

*“While TCM culture heavily influences our dietary habits like the regular consumption of Chinese herbal soups, its’ influence on healthcare service choice is unclear. When it comes to cancer management, I think both healthcare professionals, caregivers or patients have no idea about what exactly constitutes integrative oncology and how we can provide or use such services.”* – a nurse without IO experience.

*Key factor identified only in Hong Kong: lack of knowledge among healthcare professionals regarding herb–drug interactions and herbal toxicities.* From the interviews, we identified that, regardless of IO experience, participants strongly expressed that lack of knowledge among healthcare professionals (BMDs in particular) regarding herb–drug interactions and herbal toxicities is a significant implementation barrier. Healthcare professionals are worried about the potential harms incurred to patients during the concurrent use of Chinese herbs and conventional cancer treatments like chemotherapies. However, patients and caregivers are less concerned, and they may not disclose their concurrent use of Chinese herbal medicine to avoid opposition from their BMDs.

*“In Clinical Oncology settings, most, if not all, oncologists oppose the oral administration of Chinese herbal medicine during anticancer treatments. There is a lack of safety data regarding the potentially harmful interactions between concurrent oncology and herbal treatments.* – a BDM with IO experience.

*“The patient must have a certain level of white blood cells to continue chemotherapies. Also, we may need to rely on granulocyte colony-stimulating factors (G-CSFs) to boost the white blood cell level. Many patients expressed that the side effects of G-CSFs, including bone pain and fever, are unbearable and this may affect chemotherapy adherence. I think Chinese herbal medicine helps us strengthen our immune functions by elevating the white blood cell level with little to no side effects. Meanwhile, I am worried about BMDs’ disapproval of using Chinese herbs during chemotherapies due to the limited evidence on harmful interactions. Or else, I would have to lie to the BMD by not telling them that the patient consumed Chinese herbs.”* – a caregiver without IO experience.

*Key factor identified in Hong Kong and overseas: lack of knowledge among healthcare professionals and healthcare administrators regarding IO. The knowledge among healthcare professionals (BMDs and nurses in particular) about IO is essential for its implementation, given their dominant roles in the Hong Kong healthcare system. Since nurses are expected to play a pivotal role in coordinating BMDs and TCMPs collaboration, and performing the instructions placed by the two parties, having specialised knowledge in TCM and IO is paramount for them to carry out these tasks with confidence. It is even expected that they will be able to spot potential herb–drug interactions by examining both conventional medicine and Chinese herbal medicine prescriptions. In Hong Kong, healthcare professionals’ scepticism towards IO due to the lack of relevant knowledge reduced BMDs’ and nurses’ willingness to participate in IO service delivery.

*“As you call it “integrative oncology”, all parties [nurses, BMDs and TCMPs] must have a common language of communication. In Hong Kong, nursing students receive training in conventional medicine only, and nurses know nothing about TCM. If they do not receive relevant training on TCM or IO, they are unable to understand what TCMPs are doing to the patients. If we want to make nurses the coordinators between BMDs and TCMPs in IO service delivery, and the implementors of IO nursing procedures, we must provide them with adequate training and ensure that they at least understand Chinese herbal prescriptions.”* – a nurse without IO experience.

##### 3.2.1.2 Behaviour change techniques addressing the key implementation factors related to capability

Education should be provided by the local authorities to improve the awareness of IO services among healthcare professionals, healthcare administrators, patients, and caregivers, as well as to increase the field knowledge among professionals and administrators. Policies should support the provision of all administrative, logistical, and practical information necessary to outpatient IO via various media, including internal newsletters, television, radio, and newspapers. Doing so would also prepare them for the delivery and utilisation of outpatient IO services and change their attitudes towards the services.

The lack of knowledge regarding herb–drug interactions and herbal toxicities among healthcare professionals should be addressed by enablement. Policies should be developed to favour the restructuring of the environment for information exchange, generation of up-to-date scientific evidence, and provision of additional technical support on prescription safety. Enabling the creation of scientific evidence is also crucial to increasing the knowledge among professionals and administrators.

#### 3.2.2 Motivation (COM-B component)

Overall, eight themes were relevant to the motivation component of the COM-B system, covering five TDF domains (beliefs about consequences; social/professional role and identity; intention; reinforcement; and emotion) (Table 4). This component included one key factor identified in Hong Kong and overseas.

**Table 4:** Recommended implementation strategies for addressing implementation factors related to motivation.

Implementation factors identified from interviews (Themes)	COM–B (TDF domain)	Intervention functions	Policy categories	Behaviour change techniques	Implementation strategies
Lack of recognition among healthcare professionals, patients, and caregivers of the evidence supporting safety and effectiveness of IO interventions^*^	Reflective motivation (beliefs about consequences)	Education	Communication/marketing	Information about health consequences	Providing all healthcare professionals with updated research evidence on IO safety and effectiveness Promoting IO services (including information on safety and effectiveness) to the public and elucidating progress of TCM modern development
Lack of knowledge among healthcare professionals about the potential ethical or legal consequences of IO service delivery		Persuasion	Regulation	Information about social and environmental consequences	Developing regulations to clarify the duties and legal responsibilities of different healthcare professionals involved in IO services
Failure among BMDs and nurses to recognise the compatibility of IO service delivery with their duties and responsibilities	Reflective Motivation (Social/professional role and identity)	Persuasion	Communication/ marketing	Framing/reframing	Organising campaigns to promote IO services as a key government policy with substantial public demand to all healthcare professionals
Lack of career prospects for TCMPs and nurses involved in IO service delivery	Reflective motivation (Intention)	Incentivisation	Legislation	Material reward	Establishing IO specialty training schemes for TCMPs and nurses, and improve their remuneration packages upon completion of training and satisfactory job performance
Lack of motivation among healthcare professionals in delivering IO service	Automatic motivation (Reinforcement)	Coercion	Regulation	Feedback on behaviour	Setting performance indicators to monitor and evaluate the number of referrals between district health centres, TCM clinics, and BMD clinics
		Coercion	Regulation	Feedback on outcome(s) of behaviour	Establishing assessment criteria to appraise the clinical effectiveness of, and patient satisfaction towards, IO services
		Incentivisation	Service provision	Feedback on outcome(s) of behaviour	Providing reports on clinical effectiveness and patient satisfaction to healthcare professionals involved in IO services
Negative feelings among BMDs and nurses towards TCM and TCMPs	Automatic motivation (Emotion)	Persuasion	Communication/marketing	Information about social and environmental consequences	Elucidating TCM modern development to all conventional healthcare professionals with an aim to change their negative impressions towards TCM and TCMPs
Feelings of being excluded by the conventional medicine-dominated healthcare system among TCMPs		Environmental restructuring	Environmental/social planning	Restructuring the physical environment	Establishing a TCM and integrative medicine clinical governance centre supported with a multidisciplinary team of experts to advise on TCM and IO development
Lack of emotional understanding among BMDs and nurses towards patients’ requests for IO referral		Persuasion	Communication/marketing	Credible source	Inviting patients and caregivers who used IO services to share their experience with conventional healthcare professionals

BMD, Biomedically-trained doctor; COM–B, Capability, Opportunity, Motivation, and Behaviour; IO, Integrative oncology; TCM, Traditional Chinese Medicine; TCMP, Traditional Chinese Medicine practitioner; TDF, Theoretical Domains Framework.

* Themes that have a strong positive/negative influence on outpatient IO implementation and were identified in a mixed-methods systematic review (22) of international experiences on IO service implementation factors in cancer care settings (i.e., “key factors identified in Hong Kong and overseas”).

##### 3.2.2.1 Beliefs about consequences

*Key factor identified in Hong Kong and overseas: lack of recognition among healthcare professionals, patients, and caregivers on the evidence supporting safety and effectiveness of IO interventions. TCM has not been studied using modern research methodologies until recent decades. BMDs and nurses in Hong Kong may not oppose the adoption of IO, but they always expect IO interventions to be supported by robust clinical evidence, especially on safety and effectiveness. Meanwhile, patients and caregivers, who are interested in IO, also want to gather evidence to convince their BMDs to collaborate with TCMPs, with the expectation that partnership between their BMDs and TCMPs would optimise outcomes.

*“We collaborated with University X [a university in Hong Kong] on a clinical trial for assessing the effectiveness of TCM in cancer management. After several years of follow-up, we submitted the results to the oncologists in Hospital Y [a hospital in Hong Kong] for their reference. When they saw the results, they were like: “Oh! The evidence is objective.” They then started believing in TCM and initiated collaboration with us. Therefore, I think we need to go through a process of generating and promoting TCM clinical evidence, to let BMDs understand TCM and IO better. It is not only about TCMPs advocating “integration” and then BMDs will be happy to team up with us. We need to build trust.”* – a healthcare administrator with IO experience.

##### 3.2.2.2 Behaviour change techniques addressing the key implementation factor related to motivation

Similarly, the intervention function of education should be adopted by the local authorities to maximise the availability and accessibility of the latest clinical evidence on IO safety and effectiveness among both service providers and users. Policies should support the dissemination of the information via various media, including internal newsletters, television, radio, and newspapers. Doing so would help the providers recognise the evidence base of IO and the users realise the development of TCM and IO.

#### 3.2.3 Opportunity (COM-B component)

Overall, five themes were relevant to the motivation component of the COM-B system, covering two TDF domains (environment and resources; and social influences) (Table 5). This component included one key factor identified in Hong Kong and overseas and one key factor identified only in Hong Kong.

**Table 5:** Recommended implementation strategies for addressing implementation factors related to opportunity.

Implementation factors identified from interviews (Themes)	COM–B (TDF domain)	Intervention functions	Policy categories	Behaviour change techniques	Implementation strategies
Lack of nursing and administrative workforce supporting IO service delivery^#^	Physical opportunity (Environment and resources)	Enablement	Environmental/social planning	Restructuring the physical environment	Recruiting specialist nurses to coordinate care in IO services Recruiting administrative staff to assist in the operation of IO services
Lack of structured referral mechanisms and clear operation procedures for IO service delivery		Enablement	Environmental/social planning	Restructuring the physical environment	Using district health centres to coordinate IO services among TCM clinics and BMD clinics Developing formal referral mechanisms between district health centres, TCM clinics, and BMD clinics Developing clinical pathways and streamlined service processes for healthcare professionals involved in IO services Setting performance indicators to evaluate the compliance with clinical pathways and service process compliance Promoting implementation by integrating clinical pathways and service processes into existing electronic health record systems
Cost of IO services		Enablement	Fiscal measures	Social support (practical)	Formulating various patient subsidy schemes for IO services to improve access Expanding the coverage on IO services in the health insurance plans
Inefficient interprofessional communication and collaboration system^*^	Social opportunity (Social influences)	Enablement	Environmental/social planning	Restructuring the physical environment	Developing a shared electronic health record system for all healthcare professionals involved in IO services Organising team-building activities for all healthcare professionals involved in IO services

BMD, Biomedically-trained doctor; COM–B, Capability, Opportunity, Motivation, and Behaviour; IO, Integrative oncology; TCM, Traditional Chinese Medicine; TCMP, Traditional Chinese Medicine practitioner; TDF, Theoretical Domains Framework.

* Themes that have a strong positive/negative influence on outpatient IO implementation and were identified in a mixed-methods systematic review (22) of international experiences on IO service implementation factors in cancer care settings (i.e., “key factors identified in Hong Kong and overseas”).

# Themes that have a strong positive/negative influence on outpatient IO implementation but were not identified in the mentioned mixed-methods systematic review (i.e., “key factors identified only in Hong Kong”).

##### 3.2.3.1 Environment and resources

*Key factor identified only in Hong Kong: lack of nursing and administrative workforce supporting IO service delivery. A majority of participants agreed that lacking nursing and administrative workforce is a major implementation barrier for IO. Echoing the key factor of the need of training specialised IO nurses, some expressed that the availability of nurses and administrative assistants with IO knowledge and skills will be a critical factor for the successful launch or scaling up of IO services.

*“Of course, we need some more nurses for IO service delivery. However, most of the nurses in Hong Kong are only familiar with conventional medicine. You must provide them with specialised TCM training to equip them with IO knowledge and skills. Is there a role for TCM nurses? I am not sure. There is a need to find out what their competencies should be. Also, these specialised nurses should be more confident in triaging patients to IO services and introducing the details to patients. Without extra human resources, we must use our existing nurses who have already been occupied by heavy clinical and administrative tasks. If it is the case [i.e., no extra human resources], I think it is not ideal.”* – a BMD with IO experience.

*“Sometimes, giving us extra funding is not as useful as one imagines. All they [healthcare administrators] want is trained human resources. After all, they are going to hire people with the funding to help share nurses’ clinical tasks. There are also many administrative tasks required for a single IO referral. I know it is difficult to rely on those without medical backgrounds to perform clinical tasks, yet at least you can give them some administrative assistants to handle routine non-clinical tasks. I am not sure if BMDs would be happy to deliver IO services with an increased salary. However, I know they will be happy if you provide extra human resources.”* – a healthcare administrator without IO experience.

##### 3.2.3.2 Social influences

*Key factor identified in Hong Kong and overseas: inefficient interprofessional communication and collaboration system. Well-organised interprofessional communications help blur the borders between healthcare professionals and facilitate their collaborations. For instance, BMDs, TCMPs, and nurses may discuss whether the patient is eligible for IO services and whether they should be aware of any special concerns during IO treatment provision. Such communications may be realised by interdisciplinary meetings in specific and protected timeslots. However, when meetings are infeasible, shared electronic health record systems, are key to improving IO service quality as all parties would at least have access to records of all the treatments the patients are receiving.

*“Whenever patients and caregivers visit our TCMP, we need to carry around a big binder containing all records of all the conventional treatments the patient is receiving currently, and in the past. Also, we need to jot down everything the oncologist said in every consultation for our TCMP’s reference. It will be much better for all parties if the BMDs and TCMPs have some kind of an online collaboration platform for interprofessional communications.”* – a caregiver with IO experience.

##### 3.2.3.3 Behaviour change techniques addressing the key implementation factors related to opportunity

The implementation factor of lacking nursing and administrative workforce for service delivery should be tackled through enablement. In particular, the local authorities should formulate practical policies to allow the restructuring of the environment, including increasing the numbers of specialist nurses for care coordination and administrative officers for clinical operation assistance. Enablement should also be applied to improve the efficiency of interprofessional communication and collaboration. Local authorities should restructure and facilitate the interprofessional environment by, for example, establishing shared electronic health record systems and improving the morale of healthcare professionals.

## 4 Discussion

### 4.1 Comparisons with other studies on integrative medicine implementation

A qualitative study involving structured interviews and focus groups with 30 health professionals (including one acupuncturist) in the United States identified four barriers to implementing inpatient acupuncture services for pain and symptom management (36). Although acupuncture has been widely practised in the country, the uncertainty about the applicability of acupuncture, the lack of insurance coverage, time limitations, and the lack of locally relevant or setting-specific clinical evidence remained the major concerns among healthcare service providers. Another focus group study was conducted recently with 23 Canadian critical care clinicians to explore the barriers to implementing integrative therapies (i.e., natural products and mind and body practices) in adult critical care (37). The authors identified implementation barriers similar to those in our study, including the limitation of human, time, and financial resources, the lack of knowledge and training regarding relevant interventions, and the perception of lacking clinical evidence. However, they also reported that the dominance of biomedical culture was commonly mentioned by the participants, emphasising that the usual practices and priorities in intensive care units did not usually include integrative therapies. Our findings enrich the evidence base on the implementation of integrative medicine and add the perspective from a healthcare system where TCM is not officially the mainstream intervention but has been used by the population for decades.

### 4.2 Implications for local practice

When launching or scaling up outpatient IO services, Hong Kong policy-makers and authorities should endeavour to tackle the three local implementation factors (i.e., key factors identified only in Hong Kong) identified in this study as the first step. Below, we will discuss the respective potential implementation strategies targeting those factors and the possibility of applying these local implementation strategies to international contexts.

#### 4.2.1 Lack of nursing and administrative workforce supporting integrative oncology service delivery

##### 4.2.1.1 Potential implementation strategies

Care coordination is critical to the day-to-day operation of IO services because BMDs and TCMPs may not be capable of keeping track of administrative arrangements, or triaging patients for appropriate IO interventions (38, 39). To streamline IO service delivery and facilitate interprofessional collaborations, relevant local authorities can arrange additional funding to train, recruit, and retain nurses who are experienced in coordinating IO services. Besides nursing staff, administrative assistants should also be recruited or retained to handle administrative work incurred by patient referrals and triage.

#### 4.2.2 Lack of awareness of integrative oncology services among healthcare professionals, healthcare administrators, patients, and caregivers

##### 4.2.2.1 Potential implementation strategies

To build confidence among conventional healthcare professionals on the value of adding TCM to conventional care, a demonstration of how interprofessional collaboration works and how IO actually benefit patients with real-world data is essential. The public may not be familiar with current regulatory systems on TCMPs, and the malpractice complaint mechanisms in TCM practice. Such unfamiliarity may undermine the public’s confidence in TCM and IO services (40, 41). Local authorities can devolve resources into educating the public on these regulatory measures via different media. Monitoring the price transparency of TCM and IO services is also necessary if patient subsidy schemes are available in the future to facilitate access among the needy (42).

#### 4.2.3 Lack of knowledge among healthcare professionals regarding herb–drug interactions and herbal toxicities

##### 4.2.3.1 Potential implementation strategies

Local authorities can develop a publicly accessible online platform containing up-to-date knowledge on herb–drug interactions and herbal toxicities. To facilitate avoidance of negative herb–drug interactions and adverse effects, relevant evidence should be integrated into existing electronic health record systems and provide automated alerts when contra-indications or potential toxicities are detected. Pharmacists with dual qualifications in Chinese medicine and conventional pharmacy should also be recruited to help develop and update these clinical information systems regularly. They can also provide consultation services on the co-administration of Chinese herbal medicine and conventional treatment if deemed necessary in the management of complex patient cases. Real-world outcomes of co-administering Chinese herbal medicine and conventional treatment among cancer patients should also be investigated using existing data from electronic health records of the public healthcare system.

#### 4.2.4 Applying implementation strategies for the Hong Kong context to international contexts

We have located many similarities between international experiences (25) and local findings regarding IO service delivery and utilisation. The IO implementation strategies we developed for the Hong Kong context may provide policy ideas to other conventional medicine-dominated healthcare systems. From the same intervention functions and policy categories we identified, policy-makers and managers may tailor the BCTs according to their healthcare system and organisational context. For instance, a key implementation factor we identified is inefficient interprofessional communication and collaboration workflow. On top of adopting the BCT of “reconstructing the physical environment” to develop a shared information system and organise team-building activities, overseas policy-makers may also choose “goal setting (behaviour)” as an additional strategy, which may entail a minimum number of protected interprofessional meetings per month. Also, they may consider “social support (practical)” as a complementary strategy to facilitate such meetings by providing administrative assistance.

### 4.3 Implications for international practice

Given the international demands for IO services, we will discuss the potential implementation strategies that might address the key implementation factors likely to be encountered in conventional cancer care settings outside Hong Kong as well (i.e., key factors identified in Hong Kong and overseas). We will also explore the political conflicts between traditional and complementary medicine practitioners and BMDs in interprofessional collaborations and the possible solutions.

#### 4.3.1 Lack of recognition among healthcare professionals, patients, and caregivers of the evidence supporting safety and effectiveness of integrative oncology interventions

##### 4.3.1.1 Potential implementation strategies

It is vital to provide the most up-to-date clinical evidence, preferably generated using rigour clinical research methodologies, to support interprofessional decision-making and improve stakeholders’ confidence in IO service delivery (43, 44). Clinical evidence may also be incorporated into electronic health record systems with assisted decision-making functions to enhance the appropriateness and adherence (25). Similarly, clinical evidence on IO safety and effectiveness should be given to the general audience in plain language to help patients, caregivers, and the public make informed decisions on IO service utilisation (45). Appropriate dissemination of evidence will also empower them to have constructive discussions with their BMDs and TCMPs regarding their decisions to use IO services.

#### 4.3.2 Lack of knowledge among healthcare professionals and healthcare administrators regarding integrative oncology

##### 4.3.2.1 Potential implementation strategies

Healthcare professionals, especially BMDs and nurses, and healthcare administrators may not be willing to deliver IO services if they are not familiar with relevant interventions (46, 47). Therefore, authorities can arrange practical training programmes for them on a regular basis, to provide them with updated, actionable knowledge in the format of locally relevant clinical guidelines (48). Funding bodies, like the Hong Kong Chinese Medicine Development Fund (49), have been providing funding schemes to support TCM and IO research and development. Additional funding should be allocated to strengthen the creation and dissemination of IO clinical practice guidelines as this is considered to be key for evidence uptake and translation into real-world practice (50).

#### 4.3.3 Inefficient interprofessional communication and collaboration system

##### 4.3.3.1 Potential implementation strategies

Authorities can develop a shared electronic health record system for collaborative IO service delivery. This provides an efficient communication platform for BMDs, TCMPs, and nurses to exchange and update patients’ information, and spares patients and caregivers from passing information back and forth between BMDs and TCMPs (25, 42). Evidence suggests that both formal and informal bridge-building activities are effective in consolidating connectivity and fostering trust between professionals (42). Such activities may include journal clubs and mutual practice observations (51). With better professional relationships, BMD, TCMP and nurses are expected to be more willing to refer patients mutually and maintain efficient interprofessional communications (40, 52).

#### 4.3.4 Political conflicts between traditional and complementary medicine and conventional medicine

Similar to other policy areas, political conflicts are likely to be a significant factor influencing the implementation of IO (42). In healthcare systems dominated by conventional medicine, including the one in Hong Kong, the external opposition from conventional medicine regulatory bodies and the internal resistance from BMDs on the frontline are the major barriers to interprofessional collaborations (53, 54). Such conflicts could be attributed to various factors ranging from the epistemological differences between traditional and complementary medicine (e.g., TCM) and conventional medicine and the allocation of scarce healthcare resources (55, 56). It is paramount for authorities to have an effective leadership capable of creating a clear and explicit vision and direction for leading the collaborations between the two approaches, particularly in fostering consensus amongst stakeholders with backgrounds in conventional medicine (57). The key mission of the leader in these circumstances is to seek endorsement and support from stakeholders within the community and at various levels of the conventional medicine hierarchy (58, 59). Moreover, the leaders may brand the delivery of traditional and complementary medicine as a means to respond to patients’ demands for holistic care and to fill the effectiveness gaps of conventional medicine (36, 60). Relaying active “bottom-up” requests from patients may also help put interprofessional collaborations into the management agenda (61).

### 4.4 Strengths and limitations of this study

The main strength of this qualitative study is our adherence to established implementation theories in the design, analysis, and generation of implementation strategies (62). Our findings are generated from a multi-stakeholder perspective, ensuring that factors influencing outpatient IO were assessed comprehensively. We also contrasted views from stakeholders with and without IO delivery or usage experience, allowing us to pinpoint key factors relevant to launching or scaling up IO services.

This study also has several limitations. First, we did not include policy-makers in the interviews, and therefore, our implementation strategies did not consider issues from a top-down perspective. Second, we invited experienced caregivers to this study to provide insights from the users’ perspective into IO utilisation, and healthcare professionals were encouraged to share with us patients’ feedback. That said, their inputs might not entirely reflect patients’ experiences, which are critical for decision-making. Third, not all healthcare professionals who participated in this study had experience delivering IO services, the lack of relevant experience might limit their consideration of the potential factors influencing outpatient IO. Given the possibility that they could be the providers of outpatient IO, their insights and perspectives are worth exploring. Fourth, guided by the TDF and the BCW, this study focused on factors influencing outpatient IO among local stakeholders at an individual level, so the evidence generated might not be directly applicable to developing organisational change strategies. Future qualitative research is recommended to adopt the Consolidated Framework for Implementation Research (63) or other relevant theoretical frameworks to explore the barriers to and facilitators for implementing outpatient IO at an organisational level. Finally, we did not evaluate the proposed implementation strategies against the APEASE (affordability, practicability, effectiveness and cost-effectiveness, acceptability, side-effects and safety, equity) criteria. Future research is needed to refine and assess our proposed strategies in the Hong Kong healthcare system context by widening our perspectives.

### 4.5 Conclusion

In this study, we identified three common factors influencing outpatient IO service delivery and utilisation unique to the conventional-dominant healthcare system in Hong Kong: (1) the lack of nursing and administrative workforce in supporting IO service delivery, (2) the lack of awareness of IO services among healthcare professionals, administrators, patients, and caregivers, and (3) the lack of knowledge among healthcare professionals of herb–drug interaction and herbal toxicities. Implementation strategies addressing these local factors, as well as other issues identified in our study, will allow the formation of implementation strategies packages that promote the implementation of outpatient IO services in Hong Kong.

## Data Availability

The raw data supporting the conclusions of this article will be made available by the authors, without undue reservation.
