# Supplementary material for: Developing implementation strategies for promoting integrative oncology outpatient service delivery and utilisation: a qualitative study in Hong Kong
Source: Front Public Health. 2024 Aug 30;12:1414297. doi: 10.3389/fpubh.2024.1414297 (PMC11392861; doi:10.3389/fpubh.2024.1414297)
Supplement: Supplementary file 1 [file Table_1.DOCX]

Supplementary Material

**Table S1.** Definition of practices and modalities mentioned in this systematic review

**Box S1.** Interview guide for healthcare professionals with integrative oncology experience

**Box S2.** Interview guide for healthcare professionals without integrative oncology experience

**Box S3.** Interview guide for caregivers with integrative oncology experience

**Box S4.** Interview guide for caregivers without integrative oncology experience

**Table S1.** Definition of practices and modalities mentioned in this systematic review

| **Practice/modality** | **Definition** |
| --- | --- |
| Complementary medicine (CM) | - A broad set of healthcare practices that are not part of that country’s own traditional or conventional medicine and are not fully integrated into the dominant health care system ^[1]^. |
| Traditional medicine (TM) | - The sum total of the knowledge, skill, and practices based on the theories, beliefs and experiences indigenous to different cultures, whether explicable or not, used in the maintenance of health as well as in the prevention, diagnosis, improvement, or treatment of physical and mental illness ^[1]^. |
| Traditional and complementary medicine (T&CM) | - The merge of the terms TM and CM ^[1]^. |
| Integrative medicine | - A practice of medicine that selectively incorporates elements of T&CM into comprehensive treatment plans alongside solidly orthodox methods of diagnosis and treatment ^[2]^. |
| Integrative oncology | - A sub-field of integrative medicine ^[3]^. - A patient-centred, evidence-informed field of cancer care that utilises **mind and body practices**, **natural products**, and/or lifestyle modifications from different traditions alongside conventional cancer treatments ^[3]^. - It aims to optimise health, quality of life, and clinical outcomes across the cancer care continuum and to empower people to prevent cancer and become active participants before, during, and beyond cancer treatment ^[3]^. |
| Mind and body practices | - Including, but are not limited to, yoga, meditation, music therapy, acupuncture, qigong, massage (including reflexology and healing touch), and stress management ^[4]^. |
| Natural products | - Including, but are not limited to, vitamins, minerals, botanicals, and fish oil ^[4]^. |

*Retrieved from Kwong MH, et al. Integrative oncology in cancer care - implementation factors: mixed-methods systematic review. BMJ Support Palliat Care. 2023:spcare-2022-004150.*

^[1]^ World Health Organization. WHO Global Report on Traditional and Complementary Medicine 2019. Geneva, Switzerland: World Health Organization; 2019.

^[2]^ Hui D, et al. Models of integration of oncology and palliative care. Ann Palliat Med. 2015;4(3):89-98.

^[3]^ Witt CM, et al. A Comprehensive Definition for Integrative Oncology. J Natl Cancer Inst Monogr. 2017;2017(52).

^[4]^ Greenlee H, et al. Clinical practice guidelines on the use of integrative therapies as supportive care in patients treated for breast cancer. J Natl Cancer Inst Monogr. 2014;2014(50):346-58.

**Box S1.** Interview guide for healthcare professionals with integrative oncology experience

| 1. Can you share with us the first time you heard of Integrated Chinese–Western Medicine cancer palliative care services or integrative oncology? What are the facilitators of or barriers to your provision of integrative oncology services? | |
| --- | --- |
| **TDF domain** | **Question** |
| Knowledge | - Have you noticed any clinical evidence or studies on the effectiveness of integrative oncology? If yes, do you think the evidence is sufficient to prove its effectiveness? |
| Skills | - Did you need to learn any new skills or knowledge to provide integrative oncology services? - Did you attend any courses or training programmes? If yes, how important were they to your routine practice? Were they useful? |
| Intentions | - Is there any reason for you to provide integrative oncology services? - How do you feel whenever your patients or their caregivers express that they want to receive Integrative oncology services? - To what extent do you personally accept integrative oncology? |
| Goals | - If you are advising a cancer patient, do you always consider integrative oncology in your treatment? - If you want to advise cancer patients to receive integrative oncology services, what kind of obstacles will make it difficult for you? |
| Reinforcement | - How do you overcome the difficulties to provide integrative oncology services for cancer patients? - What incentives encourage you to participate in integrative oncology? |
| Behavioural regulation | - Do you have a set of criteria to help you decide whether to recommend integrative oncology services? - Do you think there is anything affecting your judgment on whether to provide integrative oncology services? - Have you and your colleagues ever discussed on the implementation of integrative oncology to reach consensus? |
| Memory, attention, and decision processes | - How consensus on treatment plan in the process of integrative oncology was reached among different clinicians? - Do you think your colleagues affect your judgment on whether to provide integrative oncology services? |
| Social/professional role and identity | - Do you think your participation in integrative oncology services affects how you view your professional status? How does it affect? - Do the views of your colleagues affect your involvement in integrative oncology? |
| Social influences | - To your knowledge, what are your supervisors’ views on integrative oncology? - Do you think your patients or their caregivers have fostered or hindered you to participate in integrative oncology? |
| Beliefs about capabilities | - Do you think it is easy or difficult to perform integrative oncology? How easy or difficult is it? - What do you think will make it easier to provide integrative oncology services? |
| Optimism | - What results do you expect from integrative oncology? Good or bad? |
| Beliefs about consequences | - Do you believe that integrative oncology makes a difference in the current cancer treatment? - Do you think the benefits of Integrative oncology are greater than the costs of doing it? - Do you think integrative oncology has brought any changes to your daily work? - What do you think would be the benefits or losses without integrative oncology? |
| Emotion | - When you see cancer patients who are in pain or desperate, does it affect your decision to provide integrative oncology services? - Do you feel stressed or worried when you provide integrative oncology services? |
| Environmental context and resources | - Do you think you there are enough resources and support to help you provide integrative oncology services? - Does your institution have any influence on your involvement in integrative oncology? |
| 1. Do you have any other ideas you would like to share with us about integrative oncology, or anything you would like to add?   **Thank you for your time and comments!** | |

**Box S2.** Interview guide for healthcare professionals without integrative oncology experience

| 1. Have you ever heard of Integrated Chinese–Western Medicine cancer palliative care services in Hong Kong?    1. If yes, under what circumstances did you hear about it? How do you understand this treatment method? 2. Have you ever thought of providing integrative oncology services?    1. If yes, are there any reasons or difficulties that make you not to provide integrative oncology services?    2. If no, what are the reasons that you do not want to participate in this service? 3. Are there any colleagues or friends of yours who provide integrative oncology services?    1. If yes, what do you think about them doing this?    2. Does the fact that your colleagues or friends are integrative oncology services makes you more interested in it? Do you think they have any influence on you? | |
| --- | --- |
| **TDF domain** | **Question** |
| Knowledge | - Have you noticed any clinical evidence or studies on the effectiveness of integrative oncology? If yes, do you think the evidence is sufficient to prove its effectiveness? |
| Skills | - Do you have any new skills or knowledge that you think you need to learn if you are to provide integrative oncology services? |
| Intentions | - Are there any situations where you think patients can/should receive integrative oncology services? - To what extent do you personally accept integrative oncology? |
| Goals | - If you are advising a cancer patient, do you always consider integrative oncology in your treatment? - If you want to advise cancer patients to undergo integrative oncology treatment, can you imagine what kind of obstacles will make it difficult for you? |
| Reinforcement | - How do you think you would overcome the difficulties to provide integrative oncology services for cancer patients? - What incentives do you think would encourage you to participate in integrative Oncology? |
| Behavioural regulation | - Do you think there is anything that would affect the judgment on whether to provide integrative oncology for participating Chinese medicine practitioners or doctors? |
| Memory, attention, and decision processes | - Can you imagine that in the process of integrative oncology, how consensus on treatment plan would be reached among different clinicians? What are the expected barriers? - Do you think your colleagues will affect the judgment of the Chinese/Western medicine practitioners in providing or not providing integrative oncology? |
| Social/professional role and identity | - Do you think your participation in Integrative oncology services would affect how you view your professional status? How does it affect? - Will the views of your colleagues on this treatment affect your involvement in integrative oncology? |
| Social influences | - To your knowledge, what are your supervisors' views on integrative oncology? - Do you think your patients or their caregivers have fostered or hindered you to participate in integrative oncology? |
| Beliefs about capabilities | - Do you expect that it is easy or difficult to provide integrative oncology services? How easy or difficult is it? - What do you think would make it easier to provide integrative oncology services? |
| Optimism | - What results would you expect from integrative oncology? Good or bad? |
| Beliefs about consequences | - Do you believe that integrative oncology will make a difference in the current cancer treatment? - Do you think the benefits of integrative oncology are greater than the costs of doing it? Prompt: Time cost, resources? - Do you think integrative oncology has brought any changes to your daily work? - What do you think would be the benefits or losses without integrative oncology? |
| Emotion | - When you see cancer patients who are in pain or desperate, does it affect your decision to provide integrative oncology services? - Do you feel stressed or worried when patients ask about Chinese medicine services? |
| Environmental context and resources | - Do you think you there are enough resources and support to help Chinese/Western medicine practitioners to provide integrative oncology services? - Does your institution have any influence on your involvement in integrative oncology? |
| 1. Do you have any other ideas you would like to share with us about integrative oncology, or anything you would like to add?   **Thank you for your time and comments!** | |

**Box S3.** Interview guide for caregivers with integrative oncology experience

| 1. Have you ever heard of Integrated Chinese–Western Medicine cancer palliative care services or integrative oncology?    1. As a caregiver, are you happy with the services? 2. Is there any advantage or disadvantage in the service model of Integrated Chinese–Western Medicine cancer palliative care or integrative oncology? Can you share with us any examples? 3. Do you think integrative oncology can alleviate patients’ symptoms or help them in any way? Can you share your experience? 4. Do you think integrative oncology can relieve your burden of caring for the patient? Can you share your experience? 5. Do you think integrative oncology can change the way you care for the patient? Can you share your experience? 6. Have the healthcare providers ever provided you with any recommendations or resources to help you or the patient provide cancer palliative care at home? 7. When the patient receives integrative oncology services, is there any barrier to the communication or collaboration between you and the healthcare providers? 8. Based on your experience, do you have any recommendations or suggestions for other patients and caregivers to promote the utilisation of integrative oncology in Hong Kong? 9. Do you have any other ideas you would like to share with us about integrative oncology, or anything you would like to add?   **Thank you for your time and comments!** |
| --- |

**Box S4.** Interview guide for caregivers without integrative oncology experience

| 1. Have you ever heard of Integrated Chinese–Western Medicine cancer palliative care services or integrative oncology? 2. Do you think of any advantage or disadvantage in the service model of Integrated Chinese–Western Medicine cancer palliative care or integrative oncology? Can you share with us any examples? 3. Do you think integrative oncology would alleviate patients’ symptoms or help them in any way? Can you share your experience? 4. Do you think integrative oncology would relieve your burden of caring for the patient? Can you share your experience? 5. Do you think integrative oncology would change the way you care for the patient? Can you share your experience? 6. Have the healthcare providers ever provided you with any recommendations or resources to help you or the patient provide cancer palliative care at home? 7. Do you have any other ideas you would like to share with us about integrative oncology, or anything you would like to add?   **Thank you for your time and comments!** |
| --- |
